# Effects of color variation and physiological state on ascidian microbiomes

**DOI:** 10.1002/mbo3.1405

**Published:** 2024-03-13

**Authors:** Samantha K. Morrison, Patrick M. Erwin, Susanna López‐Legentil

**Affiliations:** ^1^ Department of Biology & Marine Biology, Center for Marine Science University of North Carolina Wilmington Wilmington North Carolina USA

**Keywords:** 16S rRNA, microbial communities, phenotypic plasticity, phylogeny, tunicate

## Abstract

Ascidians, known for their color variation, host species‐specific microbial symbiont communities. Some ascidians can also transition into a nonfiltering (resting) physiological state. Recent studies suggest that the microbial symbiont communities may vary across different physiological states and color morphs of the host. The colonial ascidian, *Polyclinum constellatum*, which exhibits several color morphs in the Caribbean Sea, periodically ceases its filtering activity. To investigate if color variation in *P. constellatum* is indicative of sibling speciation, we sequenced fragments of the ribosomal 18S rRNA and the mitochondrial cytochrome oxidase subunit I genes. Additionally, we sequenced a fragment of the 16S rRNA gene to characterize the microbial communities of two common color morphs (red and green) in colonies that were either actively filtering (active) or nonfiltering (resting). Phylogenetic analyses of both ascidian genes resulted in well‐supported monophyletic clades encompassing all color variants of *P. constellatum*. Interestingly, no significant differences were observed among the microbial communities of the green and red morphs, suggesting that color variation in this species is a result of intraspecific variation. However, the host's physiological state significantly influenced the microbial community structure. Nonfiltering (resting) colonies hosted higher relative abundances of *Kiloniella* (Alphaproteobacteria) and *Fangia* (Gammaproteobacteria), while filtering colonies hosted more *Reugeria* (Alphaproteobacteria) and *Endozoicomonas* (Gammaproteobacteria). This study demonstrates that microbial symbiont communities serve as reliable indicators of the taxonomic state of their host and are strongly influenced by the host's feeding condition.

## Graphical abstract

Microbial symbiont communities are reliable indicators of the taxonomic state of their host and are strongly tied to their host's physiological condition.

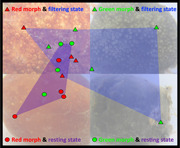

## 1 INTRODUCTION

Ascidians or sea‐squirts (Chordata; Tunicata) are sessile, benthic invertebrates characterized by a husk‐like protective casing or tunic consisting of secreted protein–cellulose complexes and sulfated polysaccharides. Considerable variation in tunic coloration exists between ascidians, even among conspecific members (Evans, Erwin, et al., 2021; Hirose et al., 2009; López‐Legentil & Turon, 2005; Tarjuelo et al., 2004). Color plasticity has been attributed to the presence of different secondary metabolites or pigments (Hirabayashi et al., 2006; Turon et al., 2005), symbiotic bacteria (Hirose et al.,^2009^), and physical structures present within the tunic (spicules; Monniot et al., 1991). Studies have also shown that intraspecific color variation had a genetic basis for some species (Evans, Erwin, et al., 2021; Tarjuelo et al., 2004) but not others (López‐Legentil & Turon, 2005). Furthermore, even within species, some colors were unambiguously linked to particular lineages, while others were not (Evans, Erwin, et al., 2021; López‐Legentil & Turon, 2005; Tarjuelo et al., 2004). For instance, López‐Legentil & Turon, (2005, 2006) found that some of the color plasticity described for the colonial ascidian *Cystodytes dellechiajei* corresponded to sibling species (i.e., blue and purple morphs), while others colors corresponded to intraspecies variation (e.g., blue, green, and white morphs). Similarly, Evans, Erwin, et al. (2021) described five color groupings for the colonial ascidian *Distaplia bermudensis* and found two distinct genetic lineages (A and B), each including 2 and 3 color groupings, respectively. Interestingly, Evans, Erwin, et al. (2021) also reported that each genetic lineage harbored a unique symbiont community.

Like all animals, ascidians form symbiotic associations with a wide range of microbes (Erwin et al., 2014; Evans et al., 2017; Tianero et al., 2015). These microbial symbionts may aid in many biological processes, including adaptation to new habitats (Casso et al., 2020; Dror et al., 2019; Evans et al., 2017), secondary metabolite production for chemical defense (Tianero et al., 2015), and protection from ultraviolet solar radiation (Hirose et al., 2004, 2006). Similar to sponges and other filter‐feeding invertebrates, ascidians acquire microbial symbionts both vertically and horizontally. Thus, some microbial symbionts are obtained directly from the progenitors (vertical transmission; Hirose, 2000, 2015; Hirose & Nozawa, 2020; Hirose et al., 2005; López‐Legentil et al., 2011, 2015; Martínez‐García et al., 2007) while others are obtained from the surrounding environment (horizontal transmission; Casso et al., 2020; Dror et al., 2019; Erwin et al., 2013; Goddard‐Dwyer et al., 2021). Despite the ability to accumulate environmentally sourced symbionts, adult ascidians harbor microbial communities that are highly host‐specific (Erwin et al., 2014; Evans et al., 2017; López‐Legentil et al., 2023) and at least in colonial ascidians, temporally stable (López‐Legentil et al., 2015).

In addition, some colonial ascidians are known to alternate between actively filtering (active) and nonfiltering (resting, dormant, or topor) phases (Turon, 1992). In colonial stolidobranch (botryllids), feeding cessation occurs regularly (e.g. weekly) and consists of resorption of the parental generation and maturation of asexually derived buds (reviewed in Manni et al., 2019). In aplousobranchs, the resting phase involves the formation of an external glassy cuticle (a thin layer of superficial tunic tissue) covering the siphonal apertures (López‐Legentil et al., 2016; Turon, 1992). Internally, the filtering apparatus (branchial sac) is reabsorbed and colonies stop filtering for a few weeks (López‐Legentil et al., 2013, 2016; Turon, 1992). This phenomenon allows for the animal's rejuvenation (Turon, 1992), the generation of new zooids after reproduction (Molin et al., 2003), or survival during periods of adverse conditions (López‐Legentil et al., 2013; Nakauchi, 1982; Pérez‐Portela et al., 2007; Turon, 1988; Turon & Becerro, 1992). Morphological and metabolic changes between active and resting forms also appeared to impact the composition of the symbiotic microbial communities. López‐Legentil et al. (2016) showed that although both filtering and resting colonies of the aplousobranch *Pseudodistoma crucigaster* maintained their core microbial symbionts, shifts in rare bacteria were detected in resting colonies, including the appearance of strictly anaerobic lineages, nitrifying bacterial guilds, and additional environmental microorganisms. Similarly, Hyams et al. (2023) found that the stolidobranch *Botrylloides leachii* harbored a resting‐specific microbiota.

The colonial ascidian *Polyclinum constellatum* (Savigny 1816) was first described from the Bermudas and is commonly observed in the Caribbean Sea (Aydin‐Onen, 2018; Lambert, 2019; Rocha & Costa, 2005; Rocha et al., 2010; Streit et al., 2021). *P. constellatum* has also been observed in the Pacific, West Indian, and Atlantic oceans (from Canada to Brazil), South Africa, and the Mediterranean Sea (reviewed in Aydin‐Onen, 2018, Virgili et al., 2022) and appears to be spreading rapidly, likely through anthropogenic vectors. Although a few barcode sequences of the cytochrome oxidase I gene (COI) are now available in GenBank (Montesanto et al., 2022; Streit et al., 2021, Virgili et al., 2022), the species has been widely misidentified in the past (Montesanto et al., 2022), presumably due to multiple color morphs occurring in sympatry (Lambert, 2019; Montesanto et al., 2022; Rocha et al., 2010; Streit et al., 2021, Virgili et al., 2022).

In this study, we used ribosomal (18 S rRNA) and mitochondrial (COI) gene fragments to determine the phylogenetic relationships between common color morphs of *P. constellatum* collected in Puerto Rico (Caribbean Sea). We also characterized the genetic profile and microbial assemblages of the two common color morphs (red and green) and two physiological states of the host (actively filtering and resting). Our goal was to determine whether (1) the observed color morphs corresponded to different genetic lineages, (2) differences in microbial communities occurred between color morphs, and (3) differences in the physiological state of the host (filtering vs. resting) altered the microbial symbiont communities in *P. constellatum*.

## 2 MATERIALS AND METHODS

### 2.1 Sample collection

**Figure 1:**
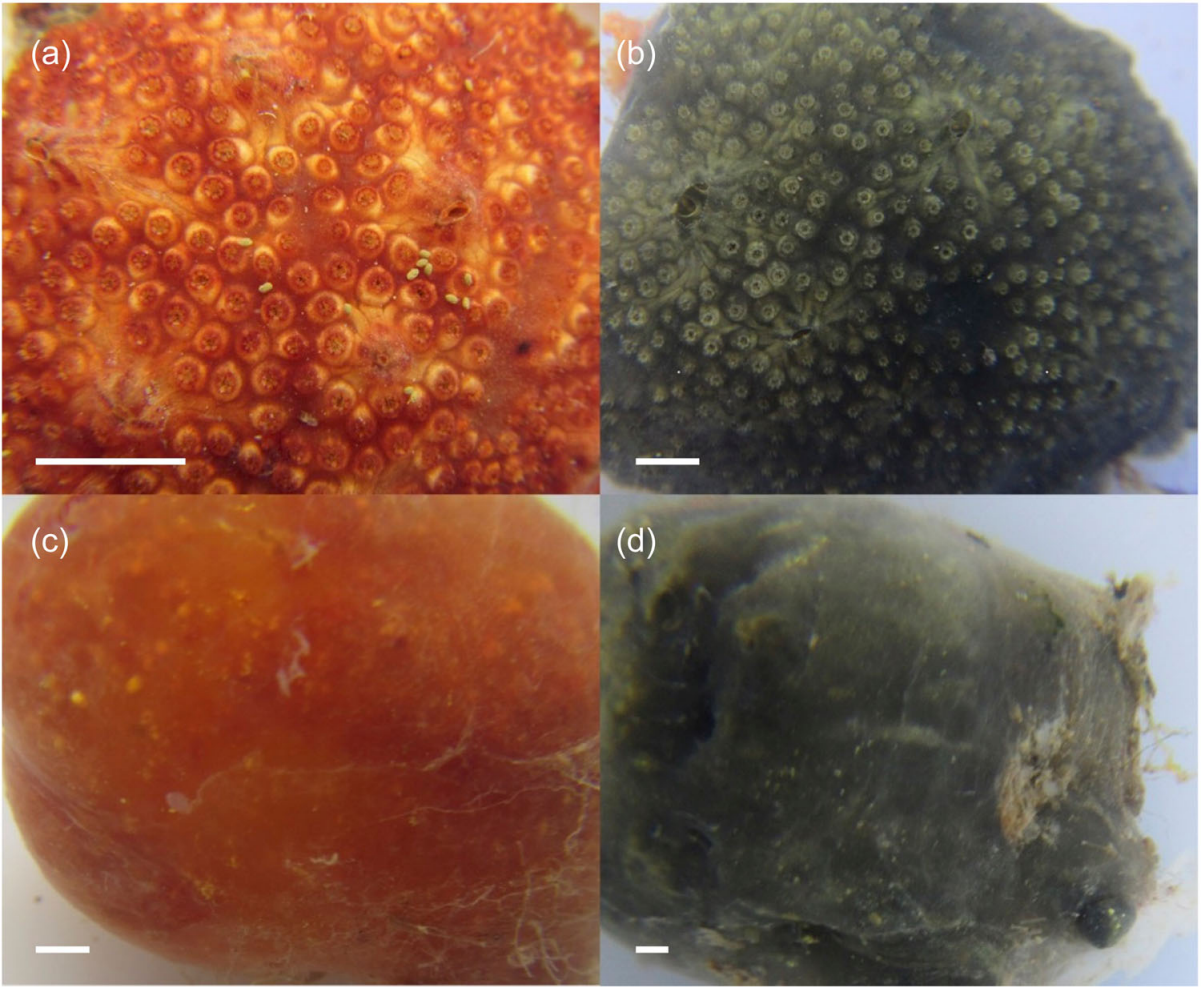
The colonial ascidian *Polyclinum constellatum* in filtering (a, b) and resting (b, c) physiological states and for the commonly encountered red (a, c) and green (b, d) color morphs in Puerto Rico. Scale bar 1 cm.

Colonies of *P. constellatum* (*n* = 17) representing two color morphs (red, *n* = 8; green, *n* = 9) and two physiological states (filtering, *n* = 9; resting, *n* = 8) were collected at a depth of 0.1–2 m from artificial substrates (boat bumpers, floating docks, and submerged ropes) in Puerto del Rey marina (18.285904, −65.636597), Puerto Rico, on February 25, 2020. Additional colonies (*n* = 43) representing various color morphs were collected from Puerto del Rey Marina (*n* = 26) and other marinas (*n* = 17) around Puerto Rico in 2019 and 2020 (Table A1). Colonies were photographed in situ and immediately placed in 20 mL vials containing absolute ethanol. External coloration and physiological state (filtering vs. resting) were recorded in situ before fixation (Figure 1).

For phylogenetic analyses, ascidians (*n* = 61) were dissected under a stereomicroscope to separate individual zooids from the tunic tissue (zooid partition). For microbiome characterization, sections of the inner tunic were dissected under a stereomicroscope using a sterile scalpel (inner tunic partition, *n* = 17). Only pieces of the inner tunic were utilized to minimize the inclusion of transient environmental microbes and epibionts.

### 2.2 COI and 18S rRNA sequencing

DNA from the zooid partition was extracted, precipitated, and purified with the DNeasy Blood and Tissue Kit (Qiagen) and stored at −20°C. A fragment (550–650 bp) of the mitochondrial COI gene was amplified with the primer set HCO2198 and LCO1490 (Folmer et al., 1994). The primer set F16 and R497 (Price et al., 2005) was used to amplify a 250–400 bp fragment of the ribosomal 18S rRNA gene. PCR amplifications were performed with a total volume of 25 μL, including 12.5 μL of MyTaq HS Red Mix DNA Polymerase (Bioline), 1 μL each of each primer, 1–3 μL of template DNA (ca. 10 ng), and purified water. The Eppendorf Mastercycler nexus X2 thermocycler conditions for COI amplification were an initial denaturation step at 95°C for 60 s; followed by 40 cycles consisting of denaturation at 95°C for 15 s, annealing at 50°C for 15 s, and extension at 72°C for 15 s; and a final extension at 72°C for 2 min. Thermocycler conditions for 18S rRNA amplification were an initial denaturation step at 95°C for 60 s; 30 cycles at 95°C for 15 s, 50°C for 15 s, 72°C for 15 s; and one final extension step at 72°C for 2 min.

PCR sequencing reactions were conducted using BigDye Terminator v. 3.1 (Applied Biosystems) and the same forward and reverse primers used for initial amplification. Sequence reactions were purified with BigDye XTerminator (Applied Biosystems), according to the manufacturer's protocol, and run on an AB 3500 genetic analyzer (Applied Biosystems) available at the Center for Marine Science of the University of North Carolina Wilmington. The raw forward and reverse sequence reads were aligned and combined into consensus sequences using Geneious version 11.1.5 (Biomatters Ltd). Sequences were obtained for 56 and 37 samples using the COI and 18S rRNA primers, respectively. All sequences are archived in GenBank with acc. numbers OQ362292–OQ362293 for the COI and OQ362356–OQ362357 for the 18S rRNA genes.

### 2.3 Phylogenetic analyses

Two phylogenetic trees were obtained for each gene analyzed. The first was constructed using the neighbor‐joining (NJ) method and the second with the maximum likelihood (ML) method. Two sequences for *Phallusia nigra* (acc. numbers KX650762 and FM244845) were obtained from GenBank and used as an outgroup for COI and 18S rRNA tree topologies, respectively. Additional sequences for the families Polyclinidae (to which *Polyclinum* belongs) and Polycitoridae (closely related; Turon & López‐Legentil, 2004) were retrieved from GenBank to build both phylogenetic trees. Due to the low number of 18S rRNA sequences available for these two families, sequences for *Distaplia* spp. (family Holozoidae) were also included to build the 18S rRNA tree. NJ trees for the COI and 18S rRNA gene fragments were conducted using the Tamura Nei (TN) method, uniform rates, and 1000 bootstraps. For ML analyses, the best evolutionary model was first determined for each gene. Analysis for the COI gene fragment was performed using the GTR + G evolutionary model and for the 18S rRNA gene we used the TN + G model. Gamma distributions were used for both models, and tree robustness was tested with 1000 bootstrap replications. Evolutionary models and trees were obtained with MEGA v.10.1.8 (Stecher et al., 2020).

### 2.4 Microbiome sample processing

To characterize the microbial symbionts, the 17 inner tunic tissue samples were sent to Zymo Research Corporation for DNA extraction, amplification, library preparation, and paired‐end amplicon sequencing (Illumina MiSeq platform) of a fragment of the16S rRNA gene (v4 region) using the primers 515F and 806R (Caporaso et al., 2011). Raw sequences were obtained and processed as described in Weigel and Erwin (2016) and detailed in Table A2, using a modified version of the Illumina MiSeq SOP pipeline in the mothur software package (v. 1.39.5; Schloss et al., 2009). In brief, raw sequences were quality filtered and aligned using the SILVA database (v. 132), the primers trimmed from the sequences, and all nontarget sequences (chloroplast, mitochondria, and eukaryotes), putative chimeric sequences (self‐reference de novo searches, UChime software package; Edgar et al., 2011) and singletons were removed (Table A2). The remaining sequences were classified using a Bayesian classifier with a bootstrap algorithm for confidence scoring (Schloss & Westcott, 2011; Wang et al., 2007), based on the SILVA reference database (v. 132). High‐quality sequences were then clustered into operational taxonomic units (OTUs) based on sequence identity (97%) and the OptiClust algorithm (Westcott & Schloss, 2017). Each OTU was assigned a taxonomic classification by majority consensus (Schloss & Westcott, 2011). Sample depth (i.e., number of sequence reads) was standardized across samples by subsampling to the lowest read count (*n* = 26,927) and all subsequent data analyses were based on these subsampled sets.

### 2.5 Symbiont community diversity and structure

Microbiome diversity and structure in *P. constellatum* were compared across the factors “color morph” (red, green) and “physiological state” (filtering, resting). The alpha‐diversity metrics OTU richness (S), inverse Simpson index (1/D), Berger–Parker index (d), and Simpson evenness index (E_1/D_) were calculated using the mothur software package (v. 1.39.5; Schloss et al., 2009). Analyses of variance (ANOVA) were used to statistically compare the diversity indices for the factors “color morph” and “physiological state” and an interaction term.

Beta‐diversity metrics were calculated based on an OTU‐dependent metric (Bray–Curtis similarity) using PRIMER (v. 7.0.13, Clarke & Gorley, 2015) and an OTU‐independent metric (weighted UniFrac distance, Lozupone & Knight, 2005) calculated in mothur. Bray–Curtis similarity matrices were based on square root transformed OTU relative abundances and visualized using nonmetric multidimensional scaling (nMDS) plots. Permutational analyses of variance (PERMANOVA) tests were used to compare microbiome similarity between the factors color morph (red, green) and physiological state (filtering, resting) and an interaction term, with the amount of variation explained by each factor calculated using estimated components of variation and significance determined using permutational *p*‐values. All significant PERMANOVA results were further analyzed with permutational multivariate analysis of dispersion (PERMDISP) to verify that all significant PERMANOVA results represented actual structural differences, rather than unequal variability of dispersion among factors. Similarity percentage (SIMPER) analyses were conducted to determine which OTUs were responsible for driving microbiome variability among factors, and metastats (White et al., 2009) was used to statistically test their differential relative abundances.

## 3 RESULTS

### 3.1 Phylogenetic analysis

**Figure 2:**
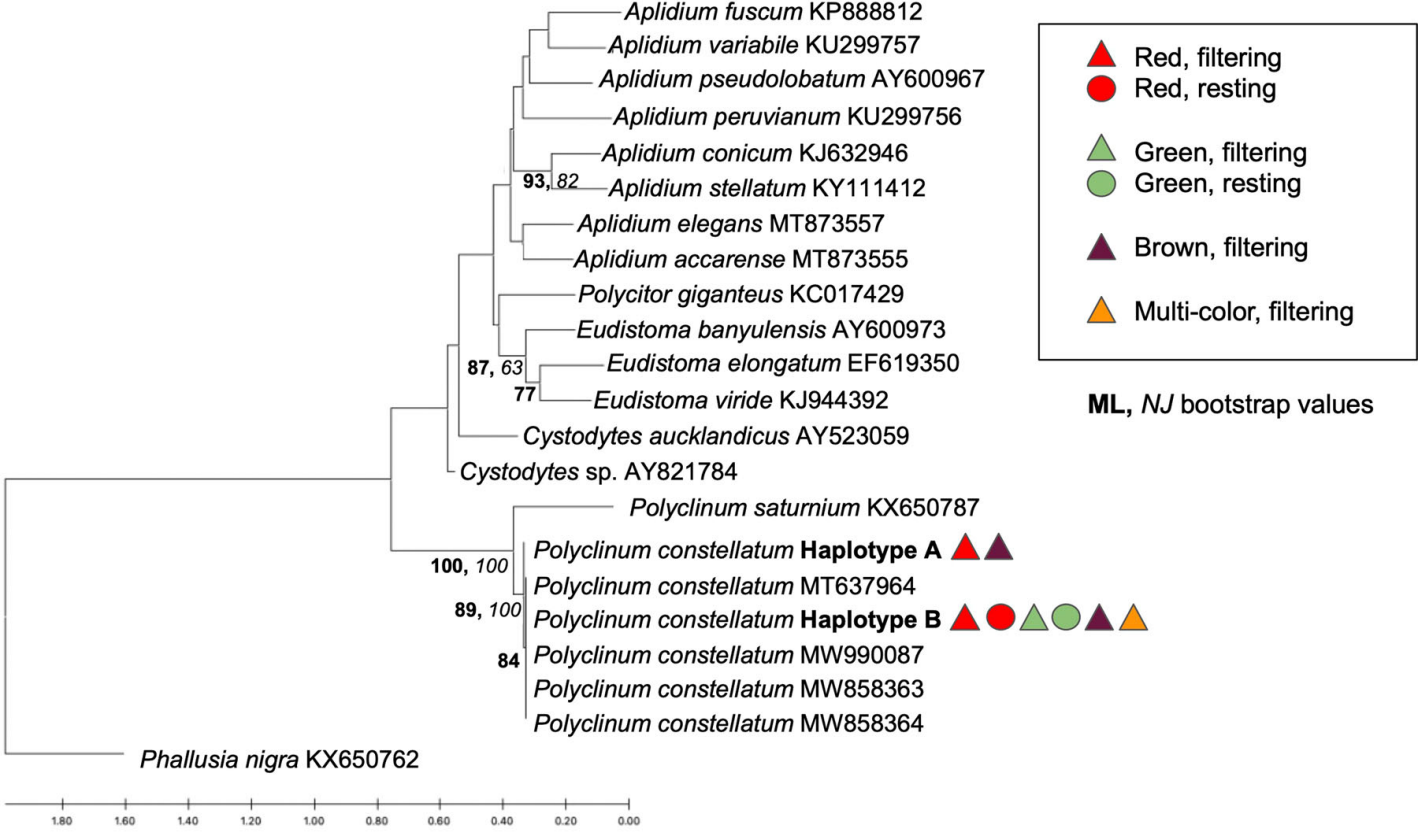
*Polyclinum constellatum* phylogeny based on partial cytochrome c oxidase subunit I (COI) sequences (*n* = 56). GenBank accession numbers follow reference sequence names in terminal node labels. Tree topology was obtained using NJ and ML analyses. Haplotype A (*n* = 3; GenBank acc. number OQ362293) contains samples 25Feb20–30, 13Mar19‐1‐19, and 8Mar19‐3‐12. Haplotype B (*n* = 53; GenBank acc. number OQ362292) consists of samples 25Feb20: 1, 4, 5, 6, 7, 8, 9, 11, 12, 13, 14, 15, 16, 17, 18, 19, 21a, 21b, 21c, 21d, 22a, 22b, 22c, 23, 24, 25, 26, 27, 28a, 28b, 28c, 29, 31, 32a, 33a, 33b, 33c, 33e; 8Mar19: 1–17, 1–25, 3–5; 10Mar19: 1–1, 1–2, 1–6, 2–10, 2–16; 12Mar19‐2‐4; 13Mar19: 1–2, 1–3, 1–4, 1–18, 1–34, 1–35. Physiological state (filtering, triangle; resting, circle) and color morph (red, green, brown, multicolored) correspond to the symbols listed after each haplotype shown. Maximum likelihood (ML) bootstrap values are shown at tree nodes in bold and neighbor‐joining (NJ) values are italicized.

**Figure 3:**
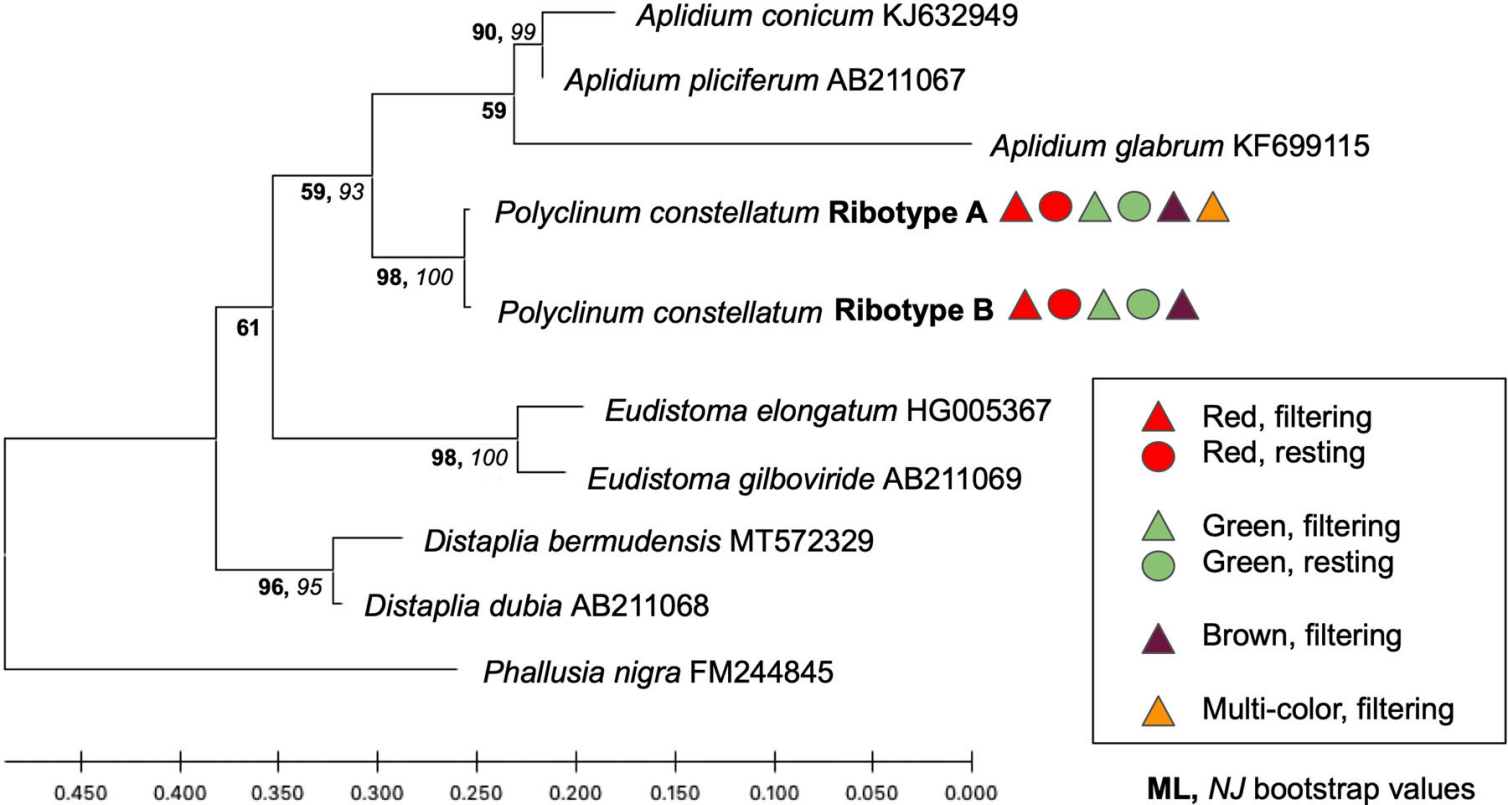
*Polyclinum constellatum* phylogeny based on partial 18S rRNA sequences (*n* = 37). GenBank accession numbers follow reference sequence names in terminal node labels. Tree topology was obtained using neighbor‐joining (NJ) and maximum likelihood (ML) analyses. Ribotype A (*n* = 20; GenBank acc. number OQ362356) included sample codes: 25Feb20 1, 7, 11, 13, 16, 17, 18, 21a, 21b, 22a, 22b, 22c, 22d, 24, 27, 28a, 29, 30, 31, 33a. Ribotype B (*n* = 17; GenBank acc. number OQ362357) includes sample codes 25Feb20 2, 3, 6, 8, 9, 12, 19, 21c, 21d, 23, 26, 28b, 28c, 33b, 33c, 33d, 33e. Physiological state (filtering, triangle; resting, circle) and color morph (red, green, brown, multicolored) correspond to the symbols listed after each ribotype shown. ML bootstrap values are shown at tree nodes in bold and NJ values are italicized.

We obtained 56 sequences for the COI gene fragment that resulted in two similar (99.5% sequence identity) haplotypes (A and B), each 621 bp. Haplotype A consisted of only three samples (codes: 8Mar19‐3‐12, 13Mar19‐1‐19, 25Feb20‐30), corresponding to two red, filtering individuals and one brown, filtering individual. The remaining 53 samples were identified as haplotype B. Only three mutations differentiated haplotypes A and B, all representing transitions (two C/T and one A/G) resulting in synonymous changes. Phylogenetic analyses using both the ML and NJ methods showed that all sequences obtained here and retrieved from GenBank for *P. constellatum* were grouped in the same clade (Figure 2) with 100% bootstrap support for both inference methods. For the 18S rRNA gene fragment, we obtained 37 sequences of 410–413 bp corresponding to two similar (98.54%) ribotypes (named A and B, respectively). Ribotype A was obtained for 20 samples and ribotype B for 17 samples. Differences between ribotypes included two mutations (a transition A/G and a transversion G/C) and one 4 bp indel that was present in ribotype A and absent in ribotype B. Phylogenetic analyses grouped all samples for all colors in a single, well‐supported clade (98% bootstrap support for the ML analysis and 100% for the NJ analysis; Figure 3).

### 3.2 Microbial assemblage structure and diversity

**Figure 4:**
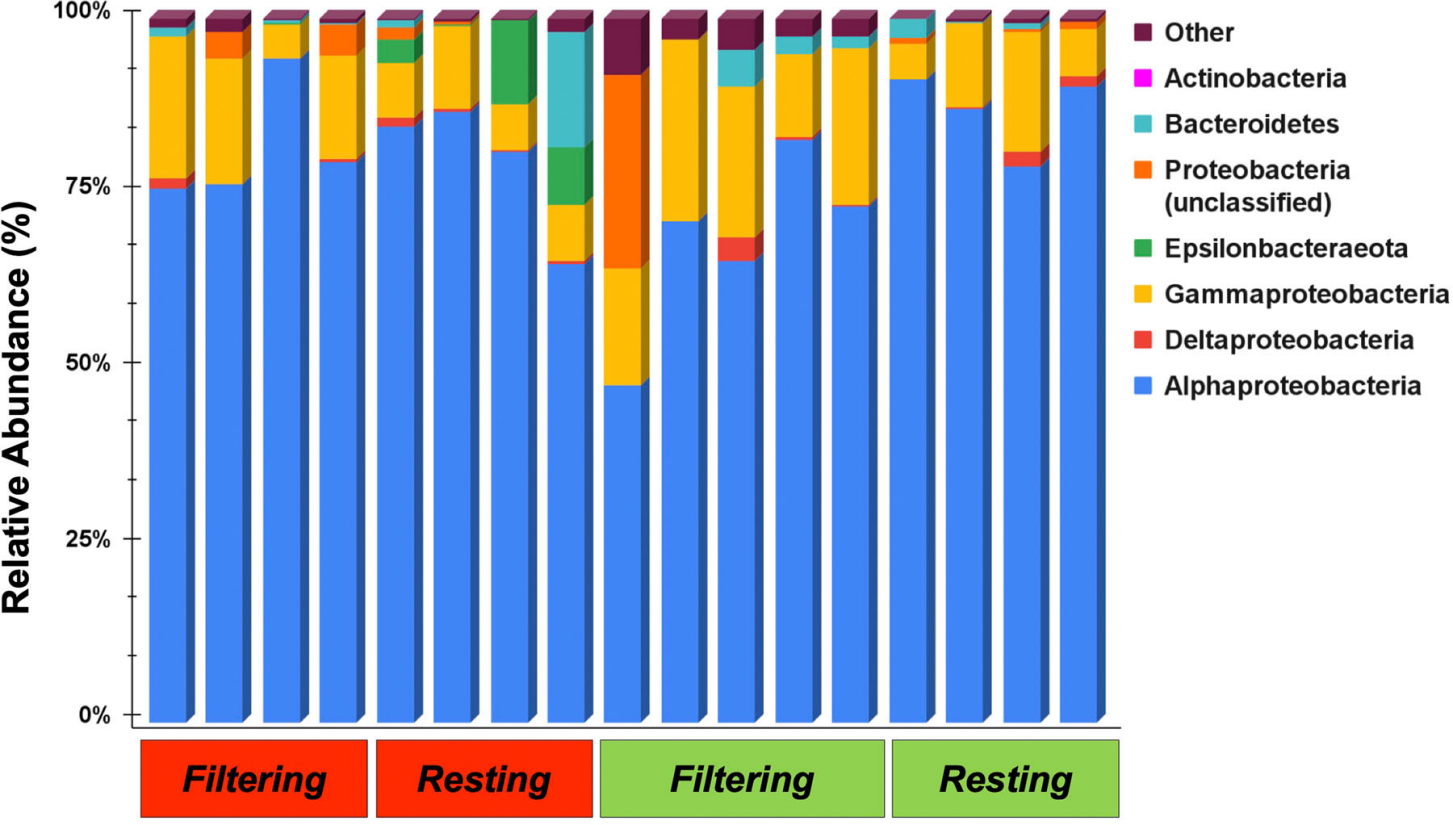
Taxonomic composition of microbial symbiont communities in *Polyclinum constellatum* grouped by host color morph (red, green) and physiological state (filtering, resting). Classifications are shown at the phylum level (Actinobacteria, Bacteroidetes) or class level (within the phylum Proteobacteria). The classification of “Other” is comprised of the rare (<1% relative abundance) phyla: Verrucomicrobia, Thaumarchaeota, Tenericutes, Planctomycetes, Patescibacteria, Omnitrophicaeota, Nitrospirae, Elusimicrobia, Dependentiae, Dadabacteria, Cyanobacteria, Chloroflexi, Acidobacteria, Acetothermia, PAUC34f, Bacteria_unclassified, and unknown_unclassified.

Microbial assemblage analyses of 17 inner‐tunic tissue samples of *P. constellatum* yielded a total of 1,392,434 high‐quality sequences that grouped into 681 unique microbial OTUs and represented 27 bacterial phyla. Proteobacteria (average relative abundance = 94.0%) was the most dominant phylum, present in all samples and composed of four main classes: Alphaproteobacteria (82.7%), Gammaproteobacteria (14.2%), unclassified Proteobacteria (2.4%), and Deltaproteobacteria (0.7%; Figure 4). Also prevalent in most samples were the phyla Bacteroidetes (1.9%, 13 of 17 individuals), Actinobacteria (1.2%, 16 of 17 individuals), and Epsilonbacteraeota (1.4%, 7 of 17 individuals). The remaining 23 phyla were rare (each <1.0% relative abundance) and together accounted for 1.6% of the overall microbial community (Figure 4).

Microbial communities associated with *P. constellatum* averaged an observed richness of 81.2 ± 15.3 OTUs (±SE) and were characterized by low evenness and high dominance of one or a few symbiont OTUs (Table 1). Statistical analysis of alpha‐diversity metrics (S, E_1/D_, d, and 1/D) revealed no significant differences in richness or evenness between color morphs or filtering states of *P. constellatum* nor an interaction term of both factors (Table A3). Red colonies exhibit higher mean richness (101.0 ± 31.8) than green colonies (64.0 ± 3.9); however, these differences were driven largely by two red colonies (one filtering and one resting) with exceptionally rich microbial communities (312 and 128, respectively). Similarly, filtering colonies averaged higher richness (88.4 ± 28.4) than resting colonies (73.0 ± 8.8), though this difference was again not significant (Table A3).

**Table 1:** Alpha‐diversity metrics for microbial communities associated with the red and green morphs and filtering and resting phases of *Polyclinum constellatum*.

Factor	S	E_1/D_	d	1/D
Red, filtering	120.75 (±64.6)	0.033 (±0.010)	0.680 (±0.107)	2.388 (±0.736)
Red, resting	80.25 (±16.43)	0.044 (±0.007)	0.476 (±0.058)	3.333 (±0.545)
Green, filtering	62.60 (±4.71)	0.048 (±0.008)	0.565 (±0.078)	3.019 (±0.532)
Green, resting	65.75 (±7.39)	0.041 (±0.006)	0.561 (±0.048)	2.617 (±0.288)

*Note*: Observed richness (S), Simpson evenness (E_1/D_), Berger–Parker index (d), and inverse Simpson index (1/D) data are shown for each factor (average ±1 SE). No significantly different means were found among color morphs and physiological states.

**Figure 5:**
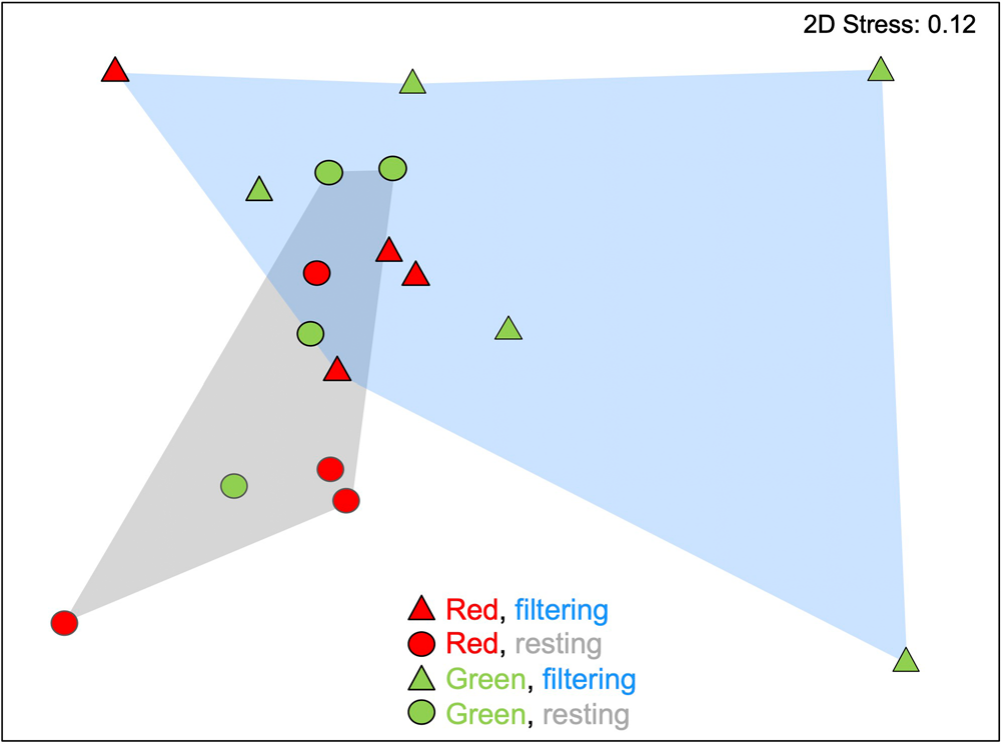
Nonmetric multidimensional‐scaling (nMDS) plot based on Bray–Curtis similarity of microbial communities in *Polyclinum constellatum*. Symbols are coded by host color morph (red, green) and physiological state (filtering = triangles, resting = circles), with polygons encompassing all filtering colonies (blue shading) and resting colonies (gray shading).

Microbial community structure was found to differ significantly between actively filtering and resting colonies of *P. constellatum* (PERMANOVA, Bray–Curtis similarity *p* = 0.047; UniFrac distance, *p* = 0.048 Table 2; Figure 5), while no significant differences were found between color morphs or the interaction term (Table 2). Accordingly, physiological state accounted for 19.2% of the observed variation in microbial community similarity, while color morph explained no additional variation and the interaction term an additional 17.9%. Multidimensional scaling plots showed tighter clustering of microbial communities in resting colonies and greater dispersion in filtering colonies (Figure 5), although these differences in assemblage dispersion were not significant (PERMDISP, *p* = 0.199). Similarly, no significant differences in dispersion were reported between microbial communities in different color morphs (*p* = 0.222, Table 2).

**Table 2:** Statistical comparisons of microbial community structure (permutational analysis of variance, PERMANOVA) and dispersion (PERMDISP) between color morphs and physiological states of *Polyclinum constellatum*.

Test	Factor	*p*‐value (Bray–Curtis)	*p*‐value (UniFrac)
PERMANOVA	Morph	0.520	0.478
	State	0.047*	0.048*
	Morph × State	0.191	0.146
PERMDISP	Morph	0.222	0.476
	State	0.199	0.942

*Note*: Results based on an OTU‐dependent metric (Bray–Curtis similarity) and an OTU‐independent metric (UniFrac distance) are shown. Asterisks (*) denote significant outcomes (*p* ≤ 0.05).

SIMPER analysis identified the main symbiont OTUs driving the significant differences observed in overall microbial assemblage structure between filtering and resting colonies. Microbial communities in resting and filtering colonies of *P. constellatum* exhibited a mean dissimilarity of 62.1%, with 10 OTUs accounting for 38.0% of this observed dissimilarity (Table 3). All 10 OTUs were members of the phylum Proteobacteria and matched closely to sequences previously reported from host‐associated and environmental sources (Table 3). OTU 2 was identified as *Kiloniella* sp. (Alphaproteobacteria) and was the main dissimilarity contributor (8%), with significantly lower mean relative abundance in filtering colonies (2.3%) than in resting colonies (21.4%). OTU 1 corresponded to *Ruegeria* sp. (Alphaproteobacteria) and contributed to 6.6% of the observed dissimilarity (Table 3), exhibiting higher abundance in filtering colonies (51.4%) than in resting ones (47.4%). OTUs 3 and 4 also corresponded to Alphaproteobacteria and both matched most closely to environmental samples (Table 3). OTU 7 was not classifiable below the phylum level and OTUs 8, 10, 5, and 6 belonged to the class Gammaproteobacteria and included species of *Endozoicomonas* (OTU 8), *Fangia* (OTU 10), *Pseudomonas* (OTU 6), and *Klebsiella* (OTU 9; Table 3).

**Table 3:** The 10 dominant operational taxonomic units (OTUs) mean abundance (mean abund.) and percent contribution (% cont.) to microbial symbiont community dissimilarity (SIMPER) between filtering and resting colonies of *Polyclinum constellatum*.

		Mean abund. (%)				
OTU ID	% Cont.	Filtering	Resting	Phylum	Lowest classification	BLASTn (% identity and accession numbers)
2*	8.04	2.34	21.35	Proteobacteria	(G.) *Kiloniella*	Sponge (100%, KF282433)
1	6.55	51.38	47.43	Proteobacteria	(G.) *Ruegeria*	Sponge and sediment (100%, MT464643; 100%, MT525294)
4	4.72	7.37	5.77	Proteobacteria	(C.) Alphaproteobacteria	Fish gut, biofilm glass (100%, KT952720; 100%, JF261920)
3	4.21	10.07	6.65	Proteobacteria	(F.) Hyphomonadaceae	Biofilm (95.22%, LT800423)
7	2.77	3.71	0.43	Proteobacteria	(P.) Proteobacteria	Gill chiton (91.16%, HE663396)
8	2.50	2.55	2.41	Proteobacteria	(G.) *Endozoicomonas*	Coral reef ecosystems (99.6%, MG896199)
10*	2.45	0.00	2.97	Proteobacteria	(G.) *Fangia*	Surface marine water (100%, NR_041041)
5	2.36	5.13	3.93	Proteobacteria	(C.) Gammaproteobacteria	Uncultured bacteria (96.02%, HQ288717)
6*	2.33	3.44	1.12	Proteobacteria	(G.) *Pseudomonas*	Roots (100%, OK039351)
9*	2.10	2.20	0.35	Proteobacteria	(G.) *Klebsiella*	Blood, human stool, human urine, soil (100%, CP083234; 100%, OK086761; 100%, OK086689; 100%, OK083774; respectively)

*Note*: Taxonomic classification of each OTU based on closest BLASTn match (G, genus; C, class; F, family; P, phylum), accession number of best match, and percent identity are also provided. Asterisks denote significant differences (Metastats, *p* < 0.05) in OTU mean abundance between filtering and resting colonies.

## 4 DISCUSSION

Color variation in the colonial ascidian *P. constellatum* did not reveal any genetic lineages. Color has been an unreliable character for species identifications in some ascidians (Bancroft, 1903; Van Name, 1945), yet, in other cases, different colorations have been linked to sibling speciation (Evans, Erwin, et al., 2021; López‐Legentil & Turon, 2005, 2006; Tarjuelo et al., 2004). Thus, the reliability of coloration in ascidian identification appears to be species‐specific. Ascidian taxonomists have few available characters to identify species, besides careful observation of the animal, its zooid morphology, and when possible, the larva. Current efforts focus on barcoding unambiguously identified species using mostly the mitochondrial COI gene (e.g., see GenBank, the Barcode of Life Data Systems). However, alternative methods are needed when gene amplifications and DNA sequencing fail. Chemotyping has rarely been employed in ascidians, although it was shown to be a reliable method to discern between sibling species (López‐Legentil & Turon, 2005). More recently, the characterization of microbial symbiont communities in species complexes has accurately resolved the taxonomic status of color variable species in ascidians (Evans, Erwin, et al., 2021) and other marine invertebrates (Evans, López‐Legentil, et al., 2021; Thacker & Starnes, 2003). Research herein corroborated these findings, as the red and green morphs of *P. constellatum* were phylogenetically indistinguishable and hosted similar microbial communities.

The physiological state of the host (actively filtering or resting) did alter the composition of the microbial symbiont communities in *P. constellatum*. To our knowledge, only one previous study has characterized microbial symbiont communities in filtering versus resting ascidians (López‐Legentil et al., 2016). Similar to observations herein, microbial symbiont communities in the temperate colonial ascidian *P. crucigaster* shifted when the animal stopped filtering. Notably, López‐Legentil et al. (2016) reported the appearance of strictly anaerobic lineages, nitrifying bacterial guilds, and environmental bacteria associated exclusively with resting ascidian colonies. In our study, dissimilarity was driven by an increased relative abundance of the proteobacteria *Kiloniella* and *Fangia* in the resting forms and of *Ruegeria* and *Endozoicomonas* in the actively filtering colonies.

The genus *Kiloniella* is formed by mesophilic, chemoheterotrophic marine bacteria with an aerobic or facultatively anaerobic (nitrate as electron acceptor) metabolism (Wiese et al., 2009). The observed shift in percent mean abundance of *Kiloniella* (OTU 2) from 2.3% in filtering colonies to 21.4% in resting colonies supports its putative ability to grow at variable oxygen concentrations resulting from filtering cessation. In addition, the ability of *Kiloniella* species to reduce nitrate to nitrite suggests an important role in nitrogen metabolism (Wiese et al., 2009). OTU 10 occurred exclusively in resting colonies and the sequences obtained herein were 100% identical to *Fangia hongkongenesis*, an aerobic Gammaproteobacteria isolated from coastal seawater in Hong Kong (Lau et al., 2007). Thus, similar to López‐Legentil et al. (2016) observations, when feeding cessation occurs in *P. constellatum*, facultative anaerobes related to nitrogen metabolism thrive, and environmental bacteria not observed in filtering colonies can colonize the animal.

In actively filtering colonies, the relative abundance of the alphaproteobacterium *Ruegeria* (OTU 1) and gammaproteobacterium *Endozoicomonas* (OTU 8) were higher than in resting colonies. Except for one species, all members of the genus *Ruegeria* are marine, and several species have been isolated from marine invertebrates (Menezes et al., 2010). All species of *Ruegeria* are chemoorganotrophic aerobes and thus a decrease in the relative abundance of this symbiont could be related to decreasing oxygen concentrations in the ascidian host. *Endozoicomonas* are conspicuous chemoorganoheterotrophic Gammaproteobacteria found in a wide range of animal hosts, including coral, sponges, and fish (reviewed in Neave et al., 2016). In ascidians, *Endozoicomonas* are also prevalent (Galià‐Camps et al., 2023; Hyams et al., 2023; Schreiber et al., 2016) and phylogenetic analyses of 16S rRNA genes have revealed an ascidian‐specific clade (Schreiber et al., 2016). Members of the same ascidian‐specific *Endozoicomonas* clade were also detected in seawater, albeit at much lower abundances, suggesting a facultative symbiosis (Schreiber et al., 2016). Further analysis revealed the presence of *Endozoicomonas* microcolonies on the branchial sac of most of the ascidian species investigated (Schreiber et al., 2016). In addition, resting colonies of *B. leachii* were enriched with a novel lineage of *Endozoicomonas* that appear to occupy specific hemocytes found exclusively in resting animals (Hyams et al., 2023). Here, we observed a decrease in the relative abundance of *Endozoicomonas* in the resting forms of *P. constellatum*. This decrease was likely due to the observed reduction of the host branchial sac that results from feeding cessation and where *Endozoicomonas* are typically found since no resting‐specific *Endozoicomonas* lineage was detected. All in all, the physiological state of the host appears to play an important role in determining microbial symbiont composition, with the appearance of a cuticle over the resting colonies and modified oxygen concentrations appearing to be the main factors driving the observed shifts.

In conclusion, our study revealed that color variation observed across colonies of *P. constellatum* did not correspond to distinct genetic lineages and that different color morphs hosted similar microbial symbiont communities. Thus, data to date indicate that conspecific ascidians harbor similar microbial communities where color variants represent the same species (e.g., *P. constellatum*, herein) and divergent symbiont communities where they represent distinct host lineages (e.g., *D. bermudensis*, Evans, Erwin, et al., 2021). Furthermore, shifts in microbiome structure were detected in actively filtering versus resting colonies of *P. constellatum*, indicating that changes in the microenvironment of the host occurred with filtering cessation and altered symbiont composition, with possible functional consequences for host–symbiont interactions (e.g., nitrogen cycling). Understanding the microbiome in relation to the holobiont physiological state is key to unlocking the largely untapped potential of microbes to aid their hosts with unique metabolic products and activities.

## AUTHOR CONTRIBUTIONS

**Samantha K. Morrison**: Formal analysis (equal); visualization (lead); writing—original draft (lead). **Patrick M. Erwin**: Conceptualization (equal); methodology (equal); formal analysis (equal); validation (equal); writing—review and editing (equal); supervision (equal); funding acquisition (equal). **Susanna López‐Legentil**: Conceptualization (equal); methodology (equal); formal analysis (supporting); validation (equal); writing—review and editing (equal); supervision (equal); funding acquisition (equal); project administration (lead).

## CONFLICT OF INTEREST STATEMENT

None declared.

## ETHICS STATEMENT

None required.

## Data Availability

The data files are available from the corresponding author. All DNA sequences have been deposited in NCBI GenBank (accession numbers for 18S rRNA sequences: OQ362356‐57; COI sequences: OQ362292‐93) and SRA (16S rRNA sequences PRJNA1040865): https://www.ncbi.nlm.nih.gov/bioproject/PRJNA1040865. All other data generated and analyzed during this study are included in this published article and its appendix.

## 1

**Table A1:** *Polyclinum constellatum* collection sites in Puerto Rico, sampling dates, sample codes, and GPS coordinates for each visited harbor.

Harbor	Date	Code	GPS
Club Náutico de San Juan	August 3, 2019	8Mar19‐1	18.459829, −66.087593
San Juan Bay Marina	August 3, 2019	8Mar19‐2	18.458877, −66.089614
Cangrejos Yacht Club	August 3, 2019	8Mar19‐3	18.45689, −66.99267
Club Deportivo del Oeste	October 3, 2019	10Mar19‐1	18.099926, −67.188005
Marina Pescaderia	October 3, 2019	10Mar19‐2	18.074423, −67.188986
Club Náutico la Parguera	November 3, 2019	11Mar19‐1	17.972542, −67.039042
Ponce Yacht & Fishing Club	November 3, 2019	11Mar19‐2	17.96343, −66.618445
Gustitos Guayama Fishing Club	December 3, 2019	12Mar19‐1	17.951831, −66.183999
Club Náutico de Guayama	December 3, 2019	12Mar19‐2	17.941197, −66.20208
Puerto del Rey Marina	December 3, 2019	13Mar19‐1	18.28773, −65.63599
Puerto del Rey Marina	February 25, 2020	25Feb20	18.28773, −65.63599

**Table A2:** Mothur pipeline commands used for 16S rRNA raw sequence quality filtering, alignment to custom SILVA (v. 132) database, primer removal, and nontarget sequence removal.

Command	Input files	Settings
make.file	Fastq	Type = gz
set.directory	InputDir	Input=
make.contigs	Fasta	Trimoverlap = T, oligos = 515f‐806r.oligos, pdiffs = 4, processors = 8
screen.seqs	Fasta, group	Maxambig = 0, Maxlength = 300, minlength = 200, maxhomop = 8
unique.seqs	Fasta	None
align.seqs	Fasta, reference	reference=silva.nr_v132.V4. align
screen.seqs	Fasta, group, name	Start = 1967, end = 11,549
filter.seqs	Fasta	Vertical = T, trump=.
pre.cluster	Fasta, group, name	Diffs = 2
chimera.uchime	Fasta, count	Dereplicate = t, reference = self
remove.seqs	Fasta, count, accnos	Accnos=
classify.seqs	Fasta, count	Reference=, taxonomy=, cutoff = 60
remove.lineage	Fasta, count, taxonomy	Taxon = Chloroplast‐Mitochondria‐Eukaryota_unknown
filter.seqs	Fasta	Vertical = T, trump=.
rename.file	Fasta	New=
rename.file	Count	New=
rename.file	Taxonomy	New=
dist.seqs	Fasta	Cutoff = 0.03, processors = 8
cluster	Column, count	column = fasta_file.final.dist, cutoff = 0.03
remove.rare	List, count	Nseqs = 1, label = 0.03
classify.otu	List, count, taxonomy	Label = 0.03
get.oturep	Fasta, count, list	Method = abundance
make.shared	List, count	None
count.groups	Count	None
sub.sample	List, count	Persample = t, size = 26,927
list.otulabels	List	None
get.outlabels	Accnos, constaxonomy	None
make.shared	List, count	None

**Table A3:** Statistical comparisons of alpha‐diversity metrics for microbial communities in *P. constellatum* by host color morph (morph), physiological state (state), and both factors together (state x morph) using analyses of variance (ANOVAs).

Metric	*p‐*Value		
	State	Morph	State x Morph
OTU richness (S)	0.63	0.26	0.50
Inverse Simpson index (1/D)	0.67	0.99	0.25
Berger–Parker index (d)	0.23	0.80	0.22
Simpson evenness index (E_1/D_)	0.92	0.40	0.27
